# A sequence-based survey of the complex structural organization of tumor genomes

**DOI:** 10.1186/gb-2008-9-3-r59

**Published:** 2008-03-25

**Authors:** Benjamin J Raphael, Stanislav Volik, Peng Yu, Chunxiao Wu, Guiqing Huang, Elena V Linardopoulou, Barbara J Trask, Frederic Waldman, Joseph Costello, Kenneth J Pienta, Gordon B Mills, Krystyna Bajsarowicz, Yasuko Kobayashi, Shivaranjani Sridharan, Pamela L Paris, Quanzhou Tao, Sarah J Aerni, Raymond P Brown, Ali Bashir, Joe W Gray, Jan-Fang Cheng, Pieter de Jong, Mikhail Nefedov, Thomas Ried, Hesed M Padilla-Nash, Colin C Collins

**Affiliations:** 1 Department of Computer Science & Center for Computational Molecular Biology, Brown University, Waterman Street, Providence, RI 02912-1910, USA; 2 Cancer Research Institute, UCSF Comprehensive Cancer Center, Sutter Street, San Francisco, CA 94115, USA; 3 Chinese National Human Genome Center, North Yongchang Road, BDA, Beijing, P.R.C. 100016; 4 Shandong Provincial Hospital, JingWuWeiQi Road, Jinan, P.R.C. 250021; 5 Division of Human Biology, Fred Hutchinson Cancer Research Center, Fairview Avenue N, Seattle, WA 98109, USA; 6 The University of Michigan, Departments of Internal Medicine and Urology, E Medical Center Drive, Ann Arbor, MI 48109-0330, USA; 7 MD Anderson Cancer Center, University of Texas, Holcombe Blvd, Houston, TX 77030, USA; 8 Amplicon Express, NE Eastgate Blvd, Pullman, WA 99163, USA; 9 BioMedical Informatics Program, Stanford University, Stanford, CA 94305, USA; 10 Bioinformatics Program, University of California, San Diego, Gilman Drive, La Jolla, CA 92093, USA; 11 Lawrence Berkeley National Laboratory, Life Sciences Division, Cyclotron Road, Berkeley, CA 94720-8268, USA; 12 Lawrence Berkeley National Laboratory, Genomics Division and Joint Genome Institute, Cyclotron Road, Berkeley, CA 94720, USA; 13 BACPAC Resources Children's Hospital Oakland, 52nd Street, Oakland, CA 94609, USA; 14 Section of Cancer Genomics, Genetics Branch, Center for Cancer Research, South Drive, Bldg. 50, MSC-8010, National Cancer Institute, National Institutes of Health, Bethesda, MD 20892, USA

## Abstract

**Background:**

The genomes of many epithelial tumors exhibit extensive chromosomal rearrangements. All classes of genome rearrangements can be identified using end sequencing profiling, which relies on paired-end sequencing of cloned tumor genomes.

**Results:**

In the present study brain, breast, ovary, and prostate tumors, along with three breast cancer cell lines, were surveyed using end sequencing profiling, yielding the largest available collection of sequence-ready tumor genome breakpoints and providing evidence that some rearrangements may be recurrent. Sequencing and fluorescence *in situ *hybridization confirmed translocations and complex tumor genome structures that include co-amplification and packaging of disparate genomic loci with associated molecular heterogeneity. Comparison of the tumor genomes suggests recurrent rearrangements. Some are likely to be novel structural polymorphisms, whereas others may be *bona fide *somatic rearrangements. A recurrent fusion transcript in breast tumors and a constitutional fusion transcript resulting from a segmental duplication were identified. Analysis of end sequences for single nucleotide polymorphisms revealed candidate somatic mutations and an elevated rate of novel single nucleotide polymorphisms in an ovarian tumor.

**Conclusion:**

These results suggest that the genomes of many epithelial tumors may be far more dynamic and complex than was previously appreciated and that genomic fusions, including fusion transcripts and proteins, may be common, possibly yielding tumor-specific biomarkers and therapeutic targets.

## Abstract 2

Tumors and cancer cell lines were surveyed with end-sequencing profiling, yielding the largest available collection of sequence-ready tumor genome breakpoints and providing evidence that some rearrangements may be recurrent.

## Background

Cancer is driven by selection for certain somatic mutations, including both point mutations and large-scale rearrangements of the genome; thus, the genomes of most human solid tumors are substantially diverged from the host genome. Many copy number aberrations have been shown to be recurrent across multiple cancer samples. These recurrent copy number aberrations frequently contain oncogenes and tumor suppressor genes, and are associated with tumor progression, clinical course, or response to therapy [1]. Moreover, it is now possible to alter the clinical course of breast cancer by the therapeutic targeting of amplified ERBB2 oncoprotein [2].

Structural rearrangements, particularly translocations, are frequently observed in solid and hematopoietic tumors. In hematopoietic malignancies the importance of translocations is well established, but their biologic and clinical significance in solid tumors remains largely enigmatic because of technical difficulties and complex karyotypes that defy interpretation. Recently, a bioinformatics approach identified recurrent translocations in about 50% of prostate tumors [3]. This discovery of recurrent translocations in prostate tumors is important because it demonstrates their presence in a common solid tumor and may make possible development of tumor-specific biomarkers and drug targets. Therapeutics such as imatinib (Gleevec, produced by Novartis Pharmaceuticals, East Hanover, NJ, USA), which are are directed toward tumor-specific molecules, may be more efficacious with fewer off-target effects than therapies aimed at molecules whose structures and/or expression are not tumor specific.

**Figure 1:**
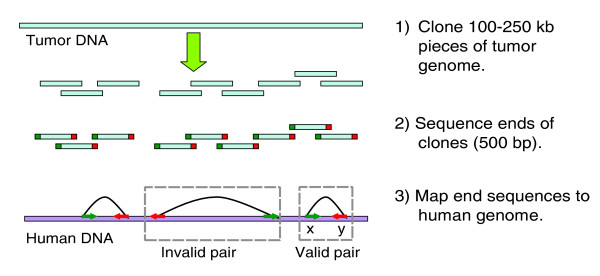
Schematic of ESP. End sequencing and mapping of tumor genome fragments to the human genome provides information about structural rearrangements in tumors. A bacterial artificial chromosome (BAC) end sequence (BES) pair is a valid pair if distance between ends mapped on the normal human genome sequence and the orientation of these ends and are consistent with those for a BAC clone insert; otherwise, the BES pair is invalid. bp, base pairs; ESP, end sequencing profiling.

End sequencing profiling (ESP) is a technique that maps and clones all types of rearrangements while generating reagents for functional studies [4-7]. To perform ESP using bacterial artificial chromosomes (BACs), a BAC library is constructed from tumor DNA, BACs are end sequenced, and the end sequences aligned to the reference human genome sequence (Figure 1). Previous ESP analysis of the breast cancer cell line MCF7 revealed numerous rearrangements and evidence of co-amplification and co-localization of multiple noncontiguous loci [6,7]. Similarly complex tumor genome structures were recently identified in cell lines derived from breast, metastatic small cell lung, lung and neuroendocrine tumor using BAC end sequencing [8].

We performed ESP on the following: one sample each of primary tumors of brain, breast, and ovary; one metastatic prostate tumor; and two breast cancer cell lines, namely BT474 and SKBR3. Hundreds of rearrangements were identified in each sample, some of which may encode fusion genes. Fluorescence *in situ *hybridization (FISH) confirmed the presence of translocations predicted by ESP in BT474 and SKBR3 cells. Sequencing of 41 BAC clones from cell lines and primary tumors validated a total 90 rearrangement breakpoints. Mapping these breakpoints in multiple breakpoint spanning clones provided evidence of numerous genomic rearrangements that share similar but not identical breakpoints, a phenomenon analogous to the inter-patient variability of breakpoint locations in many fusion genes identified in haematopoietic cancers. Comparison of rearrangements shared across multiple tumors and/or cell lines suggests recurrent rearrangements, some of which confirm or suggest new germline structural variants, whereas others may be recurrent somatic variants. Analysis of single nucleotide polymorphisms (SNPs) in BAC end sequences revealed putative somatic mutations and suggests a higher mutation rate in the ovarian tumor.

ESP complements other strategies for tumor genome analysis including array comparative genomic hybridization (aCGH) and exon resequencing by providing structural information that is otherwise not available. New sequencing technologies [9] promise to decrease radically the cost of ESP and thus make it widely applicable for analysis of hundreds to thousands of tumor specimens at unprecedented resolution. The present study previews the discoveries of such future large-scale studies, examines some of the challenges these studies will face, and provides reagents (genomic clones) for further functional studies, particularly for cell lines that have proved useful as models for cancer research [10,11].

## Results

### Tumor BAC libraries

BAC libraries were constructed from frozen samples from two breast tumors and single tumors from the brain, ovary, and prostate, demonstrating that there is no tumor-specific bias for BAC library construction. Approximately 50 mg to 200 mg of fresh frozen tumor specimen was used in the construction of each library. All tumors were dissected to minimize contamination with normal tissue. BAC libraries from the breast cancer cell lines BT474 and SKBR3 were also constructed. Breast cancer cell lines were included in this study because their genomes and transcriptomes are similar to those identified in primary breast [10,11] and are invaluable for functional studies. BT474 and SKBR3 were chosen because their aCGH profiles are similar to the profile of previously studied MCF7 cell line [6,7]. All three cell lines have very high amplifications at the *ZNF217 *locus on 20q13 and very high amplifications at chromosome 17. Table 1 lists the clinical characteristics of the tumors and properties of the BAC libraries.

**Table 1:** Clinical characteristics of the brain, breast, ovary and prostate tumor samples, and three breast cancer cell lines used for BAC library construction

	Library name							
	
	AA9	B421	CHORI-514	MCF7	PM-1	CHORI-510	CHORI-518	CHORI-520
Clinical sample designation	AA9	B421	S104	MCF-7	25-48	860-7	BT-474	SK-BR-3
Organ site	Brain	Breast	Breast	Breast cancer adenocarcinoma (metastasis - pleural effusion)	Prostate metastasis	Ovarian carcinoma	Ductal carcinoma	Breast cancer adenocarcinoma (metastasis - pleural effusion)
Therapies applied	Radiotherapy	Chemotherapy 4 months before surgery (CMF)	No radiation therapy or chemotherapy before surgery	N/A	Hormone ablation, palliative radiotherapy	No therapy before surgery	N/A	N/A
Patient status	Deceased	Deceased, no recurrence	No recurrence for 10 years	N/A	Deceased	Tumor recurred within 13 months	N/A	N/A
Total amount of tumor material used for library construction (mg)	100	150 (20 mg effective)	100	N/A	50	200	N/A	N/A
Average clone size (± standard deviation; kb)	129.1 ± 38.3	136.4 ± 29.2	166.1 ± 53.2	148.0 ± 30	N/D	149.3 ± 28.8	179 ± 23	154 ± 25

Shown are the clinical characteristics of the recurrent glioblastoma AA9, primary breast tumors B421 and S104, ovarian tumor 860, prostate metastasis 25-48, and the breast cancer cell lines MCF7, BT474, and SKBR3 used for bacterial artificial chromosome (BAC) library construction. Average clone size was determined by pulsed field-gel electrophoresis of Not1-digested DNA from 30 to 100 clones. The presence of a large blood clot in the B421 sample reduced the effective amount of tumor tissue to an estimated 20 mg (out of about 150 mg received from the tumor bank). CMF, cyclophosphamide, methotrexate and fluorouracil; kb, kilobases; N/A, number is not applicable for cell lines that can be grown in any amount and whose clinical history is not available; N/D, number not determined.

### BAC end sequencing and mapping

End sequences of 4,198 BAC clones from the brain tumor library, 5,013 clones from the metastatic prostate library, 5,570 clones from ovary tumor library, 9,401 and 7,623 clones each from primary breast libraries, 9,580 clones from the BT474, and 9,267 clones from the SKBR3 breast cancer cell lines were generated. The end sequences (59.7 megabases [Mb] in total) were mapped to the reference human genome sequence, and the results are summarized in Table 2. We analyzed end sequences that mapped uniquely to the reference sequence, excluding those in repetitive regions, segmental duplications, or duplication-rich centromeric and subtelomeric regions. The density of mapped end sequences in ESP closely matched copy number profiles generated using tiling path BAC arrays [6]. Outside these regions, the distribution of mapped end sequences along the genome did not exhibit other significant gaps or high density, arguing against any unusual cloning bias or mapping artifacts. For comparison and further analysis, we included 29.7 Mb of sequence from 19,831 end sequenced clones from MCF7 and 701 end sequenced clones from a normal human library (K0241) previously reported [7].

**Table 2:** Results of end sequencing and mapping of each library

	MCF7	BT474	SKBR3	Breast	Breast.2	Ovary	Prostate	Brain	Normal
Library name	MCF7_1	CHORI-518	CHORI-520	B421	CHORI514	CHORI510	PM1	IGBR	K0241
Mapped clones (*n*)	12,143	8,044	7,363	6,972	5,678	3,946	3,499	3,238	609
Unique mapped clones (*n*)	11,492	7,547	6,950	6,540	5,381	3,714	3,296	3,051	568
Valid pairs (*n*)	11,001	7,361	6,763	6,376	5,268	3,627	3,200	2,984	560
Contigs (*n*)	6,323	4,135	4,171	4,365	3,450	2,877	2,747	2,573	548
Contig coverage	0.324	0.327	0.274	0.233	0.243	0.155	0.104	0.103	0.019
Invalid pairs (*n*)	491	186	187	164	113	87	96	67	8
Fraction invalid	0.043	0.025	0.027	0.025	0.021	0.023	0.029	0.022	0.014
*P *value	4.10 × e^-04^	0.056	0.032	0.051	0.133	0.080	0.020	0.113	NA
Number clusters (*n*)	36	26	24	2	7	2	2	0	0
Invalid pairs in clusters (*n*)	164	61	64	4	24	4	4	0	0

The fraction of invalid pairs is calculated relative to the number of uniquely mapped pairs. The *P *value is the probability that the fraction of invalid pairs is the same as observed in the normal library, using a sample proportion test with pooled variance.

Each clone with uniquely mapped ends gives a BAC end sequence (BES) pair. A BES pair is a valid pair if distance between ends mapped on the normal human genome sequence and the orientation of these ends and are consistent with those for a BAC clone insert; otherwise, the BES pair is invalid (Figure 1). An invalid pair indicates a BAC clone that may span a genomic rearrangement. These are relatively rare, comprising 2.1% to 4.3% of the mapped BES pairs (Table 2 and Additional data file 1 [Table S1]). The largest fractions of invalid pairs are observed in the three breast cancer cell lines, with the greatest (4.3%) observed in MCF7. The majority of these invalid pairs map to amplicons known to co-localize with other loci. DNA within these structures is highly rearranged [4-7]. Among the primary tumors, the greatest fraction of invalid pairs is in the prostate metastasis library (Table 1).

For each library, we formed BES clusters grouping invalid pairs with close locations and identical orientations that are consistent with the same genome rearrangement [4]. Each BES cluster provided evidence that the inferred rearrangements are not experimental artifacts. We identified numerous BES clusters in each tumor (Table 2). The fraction of end-sequenced clones that lie in clusters is much lower for clinical tumor samples than cell lines, possibly because of the lower sequence coverage, normal tissue admixture, or greater genomic heterogeneity in the primary tumors. Moreover, the coverage of the genome by valid pairs was significantly lower than either predicted by Lander-Waterman statistics or obtained by modeling using matched *in silico *BAC libraries (see Additional data file 1 and Additional data file 2 [Figures S1 and S2]). This apparent reduction in coverage is probably a result of differing amounts of aneuploidy and genomic heterogeneity in the samples.

### Sequencing rearrangement breakpoints

We performed low coverage sequencing of 37 BAC clones corresponding to invalid BES pairs and combined these data with ten previously sequenced MCF7 BACs [7]. For each BAC, 96 3-kilobase (kb) subclones were end-sequenced, and subclones spanning the breakpoints identified. These subclones were then sequenced to pinpoint the breakpoints more precisely. This procedure identified 90 rearrangement breakpoints in 41 BACs with some BACs containing multiple breakpoints (Table 3 and Additional data file 3 [Table S2]). Breakpoints in six clones could not be identified due to repetitive elements and/or genome assembly problems (see Additional data file 1). The sequencing of these 41 clones confirmed the genomic locations of the BES determined by ESP and identified translocation breakpoints in primary tumors of the breast, brain, ovary, and a metastatic prostate tumor. In the breast cancer cell line MCF7, all clones with multiple breakpoints mapped to a highly rearranged amplicon of co-localized DNA from chromosomes 1, 3, 17, and 20, consistent with an earlier report [7] demonstrating that up to 11 breakpoints can be present in a single 150-kb clone.

**Table 3:** Summary of BAC sequencing

Sample	Clones with identified or sequenced breakpoints	Total number of identified/sequenced breakpoints	Intragenic rearrangements	Gene:intergenic fusions	Gene:gene fusions	Intergenic: intergenic fusions
MCF7	12	36/35	3	10	19	4
BT474	6	15/6	3	2	10	0
SKBR3	8	24/8	7	4	12	1
Breast (2B421)	3	3/3	0	0	3	0
Breast (CH514)	0	-	-	-	-	-
Ovary	4	4/4	0	0	4	0
Prostate	5	5/5	0	4	1	0
Brain	3	3/3	0	3	0	0

Breakpoints are indicated as sequenced if the nucleotide sequence was obtained, or identified if the breakpoint was localized to 3-kilobase subclones. BAC, bacterial artifical chromosome.

Of the 90 breakpoints identified in these 41 BACs, 63 were sequenced, and the remaining 27 were localized to 3-kb subclones. Because gross genomic rearrangements result from aberrant double strand break (DSB) repair, we analyzed the rearrangement breakpoints for signatures of the two major DBS repair mechanisms: nonallelic homologous recombination and nonhomologous end joining (NHEJ). We analyzed the repeat content and structure of the 63 breakpoint junctions, 53 of which were nonredundant (see Additional data file 3 [Table S3]). These 53 nonredundant junctions encompass 31 translocations, 12 deletions, and 10 inversions. Two junctions (representing two translocations) contain Alu elements spanning the breakpoints and are consistent with DSB repair by Alu-mediated nonallelic homologous recombination. All of the remaining junctions (51/53 [96%]) are consistent with NHEJ repair and either span microhomology regions ranging in size from 1 to 33 base pairs (45/51) or lack any homology (6/51) between the two regions involved in a particular rearrangement. We find insertions at the junction site ranging from 1 to 31 base pairs in 7 out of 51 NHEJ events. Twenty of the 106 breakpoint sites deduced from the nonredundant junction analyses are located within regions of known structural variation.

Of the 90 breakpoints, 72 are predicted to alter gene structure, resulting in either gene fusions or fusions of gene fragments to intergenic regions. This high proportion reflects a nonrandom selection of clones for sequencing, with priority given to clones that are likely to encode fusion genes [12]. Of the remaining 18 breakpoints, three indicate deletions of multiple genes. For example, a breakpoint on chromosome 17 indicates a deletion of five genes (*EFCAB3*, *METTL2A*, *TLK2*, *MRC2*, and *RNF190*). An additional seven breakpoints are located within genes and may result in intragenic rearrangements (for example, the *DEPDC6 *gene on chromosome 8). The remaining eight breakpoints are either rearrangements involving intergenic regions or microrearrangements within introns.

### Breakpoint heterogeneity

**Figure 2:**
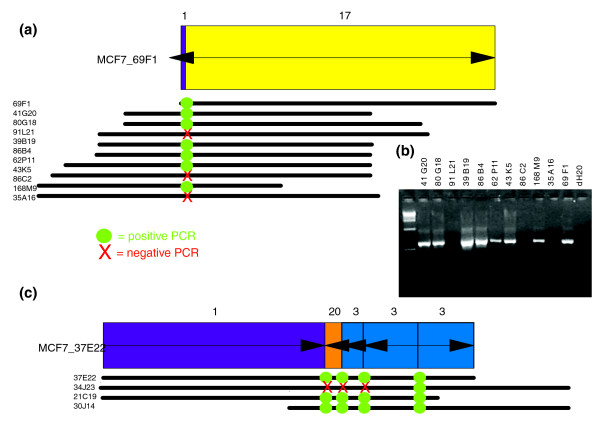
PCR validation of breakpoints in MCF7. **(a) **MCF7 clone 69F1 was sequenced and contained a small piece of chromosome 1 (purple rectangle) to chromosome 17 (yellow rectangle). Arrows on each rectangle indicate whether the fragment is oriented as in the reference genome (pointing to right) or inverted (pointing to left). PCR primers were designed to amplify the breakpoint and these primers were used to assay the other clones in the BES cluster with 69F1. Each of the other clones in the cluster are indicated as lines below 69F1, with the end-points of the lines indicating the locations of the mapped ends relative to the ends of 69F1. The heterogeneous PCR results might result from heterogeneity of the MCF7 cells, or the existence of multiple versions of this breakpoint in MCF7 genome. **(b) **PCR results for the clones presented in panel a. The expected size of the PCR fragment is 600 base pairs. **(c) **PCR validation of breakpoints in sequenced clone 37E22 from MCF7 and three additional clones in bacterial artificial chromosome end sequence (BES) cluster all fusing nearby locations from chromosomes 1, 3, and 20. Two other clones have the same complex internal organization as 37E22 with four rearrangement breakpoints. However, clone 34J23 contains only one of these breakpoints, suggesting that the rearrangement history of this clone is different from that of the others in the cluster.

BAC clones in amplicons such as those on chromosomes 1, 3, 17, and 20 in MCF7 are highly over-represented and consequently form large BES clusters of invalid pairs. Sequencing of a few of these clones [7] revealed that they often span multiple breakpoints. We assessed whether all clones in a BES cluster share the same complex internal organization by assaying the presence of sequenced breakpoints by PCR. In total, we examined 23 breakpoints in 41 clones from seven BES clusters. The majority (69/96) of the PCR assays indicated that breakpoints are shared between clones in the same BES cluster. Surprisingly five of seven BES clusters are heterogeneous in breakpoint composition, meaning that clones with nearby mapped ends do not necessarily span the same breakpoints (see Additional data file 3 [Table S4]). For example, MCF7 clone 69F1 with one sequenced breakpoint is a member of a cluster with 11 clones, but only 8 of 11 clones contain the 69F1 breakpoint (Figure 2a,b). Another clone, 37E22, was previously shown to contain four breakpoints [7]. Of the three clones in the BES cluster with 37E22, two clones contain all four breakpoints, whereas one contained only one of the breakpoints (Figure 2c). In all cases PCR validated the end locations of all negative clones, confirming the presence of alternative breakpoints in these clones. Although the mapped end sequences of the clones in these heterogeneous clusters confirmed that they fuse similar genomic loci, we hypothesize that similar rearrangements occurred in multiple copies of these loci, because of either earlier duplications in MCF7 or genomic heterogeneity in different cells in the MCF7 population. Although such variability in breakpoint location, or breakpoint wandering, is observed in fusion genes shared across multiple patients (for example, the *BCR*-*ABL *gene in leukemia [13]) and there are numerous reports of genomic heterogeneity in cell lines [14,15], this is the first time that it has been observed on a microgenomic scale within a single sample.

### Rearrangement validation

**Figure 3:**
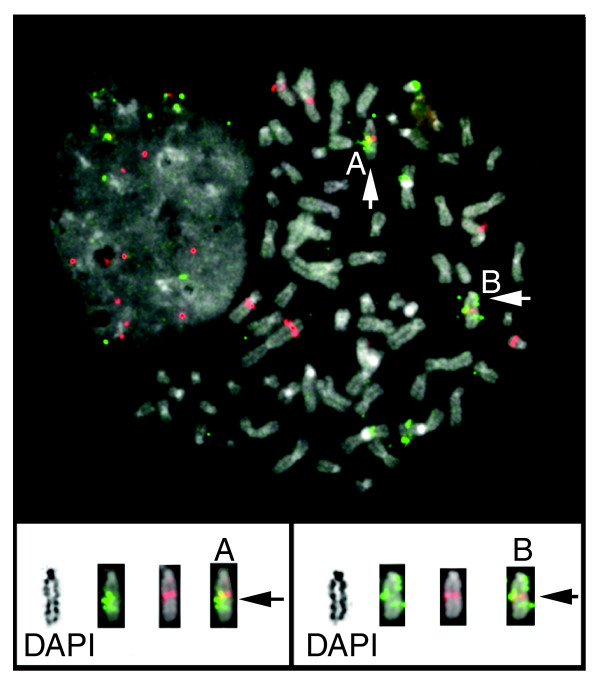
Use of dual-color FISH to validate a BT474 genomic breakpoint. End sequences from clone CHORI518_014-E04 were mapped to chromosomes 1 and 4. Clones RP11-692N22 and RP11-1095F2 were selected from the human RPCI11 library because their sequences map to just outside of tumor bacterial artificial chromosome (BAC) end sequence (BES) locations. These BACs were labeled with fluorescein and Texas red, respectively. Top: two chromosomes containing a merged yellow signal indicating juxtaposition of both probes are indicated with white arrows (and labeled A and B). Bottom: each labeled chromosome is shown with corresponding inverted-DAPI banded chromosome, and red and green image layers. Black arrows identify the region where the red and green probes are juxtaposed to one another. FISH, fluorescence *in situ *hybridization.

We validated a subset of breakpoints detected in the BT474 and SKBR3 breast cancer cell lines using dual-color FISH. Normal BAC clones were selected that flank the predicted breakpoints in the reference human genome, and FISH was performed to metaphase spreads from the cell lines. Four BT474 and two SKBR3 breakpoints were confirmed using dual-color FISH (Figure 3). In addition DNA fingerprinting was employed [16-20] on a subset of clones from the MCF7, brain, and breast (B421) BAC libraries. Excellent correlation between BES mapping and fingerprint mapping was observed; fingerprint analysis confirmed the absence of the rearrangements in 250 out of 261 (96%) BAC clones predicted not to span rearrangement breakpoints and confirmed the presence of breakpoints in 154 out of 226 (68%) clones predicted to span genomic breakpoints by ESP [21].

### Identification and analysis of recurrent breakpoints

We clustered BES pairs from all ESP datasets together and identified 62 recurrent clusters that contain BES pairs from multiple samples whose mapped ends are close. Recurrent clusters may be caused by recurrent somatic mutations, structural polymorphisms [22], mapping problems, or assembly errors in the reference genome. Most recurrent clusters (60/62) fall into two classes: mapping to pericentromeric/subtelomeric regions (9) or micro-rearrangements (56), defined here as rearrangements with breakpoints less than 2 Mb apart. Five clusters fall into both classes. For the micro-rearrangements, 21 out of 56 (38%) overlap known structural variants [23] (see Additional data file 3 [Table S5]), which is nearly a threefold enrichment over the 15% of nonrecurrent clusters corresponding to known structural variants. The remaining 35 clusters may detect novel structural variants or cancer-specific rearrangements. For example, a pericentric inversion on chromosome 11 was identified in two breast tumors and all three breast cell lines (see Additional data file 1 [Table S6]). Other examples include an 820 kb deletion in 17q23.3 in MCF7 and BT474 that contains the *TRIM37*, *GDPD1*, *YPEL2*, *DHX40*, and *CLTC *genes, and a 4 Mb deletion of gene-rich region in 10q11.22-10q11.23 in BT474 and a primary breast tumor (CHORI514; see Additional data file 1 [Table S6] and Additional data file 2 [Figure S3]).

**Figure 4:**
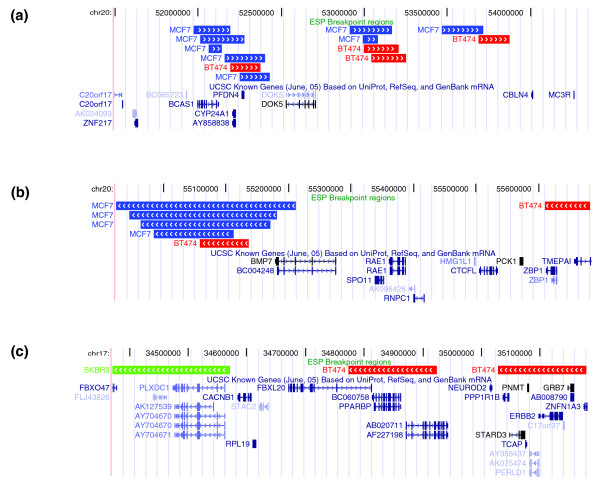
Recurrent rearrangement loci in the three breast cancer cell lines. **(a,b) **Four loci on 20q13.2-13.3 shared by MCF7 and BT474 and **(c) **a locus near to the ERBB2 amplicon shared by BT474 and SKBR3. Colored boxes indicate the breakpoint regions for different bacterial artificial chromosome (BAC) clones from MCF7 (blue), BT474 (red), and SKBR3 (green) as a custom track on the University of California, San Francisco (UCSC) genome browser. A breakpoint region is defined as the possible locations of a breakpoint that are consistent with all the BAC end sequence (BES) in the cluster; thus, shorter boxes indicate more precise breakpoint localization. Arrows give the strand of the mapped BES and thus point away from the fused region.

The largest number of BES clusters is found in the ESP datasets from the breast cancer cell lines BT474, MCF7, and SKBR3. ESP identifies known amplicons, deletions, and translocations present in these cell lines [24-26]. We searched for genomic loci that contain a rearrangement breakpoint in at least two of these three cell lines. To minimize the possibility of experimental errors, we first restricted consideration to rearrangement breakpoints identified by a BES cluster in each cell line. We identified six examples of such recurrent rearrangement loci. Four loci shared between MCF7 and BT474 map to the 20q13.2-20q13.3 amplicon and have ends clustered within 2 Mb (Figure 4a,b). It might be significant that the breakpoints in MCF7 occur in and/or truncate BCAS1, possibly explaining its total lack of expression in MCF7 cells despite being amplified [27]. In contrast, BCAS1 is highly amplified and expressed in BT474 cells [27], and the breakpoints map immediately distal to BCAS1 (Figure 4a). In addition, the regular spacing of breakpoints in this locus is suggestive of breakage/fusion/bridge (B/F/B) cycles [7]. Two additional loci are common to BT474 and SKBR3. One locus includes breakpoints that cluster within about 500 kb of the *ERBB2 *gene, which is amplified and over-expressed in these cell lines [26]. In SKBR3, these breaks co-localize the *ERRB2 *locus with an amplified region from chromosome 8 (Figure 4c). In the last example, breakpoints in BT474 and SKBR3 are predicted to disrupt the ubiquitin protein ligase gene *ITCH *at 20q11.2. When considering rearrangement breakpoints defined by all invalid pairs, rather than only BES clusters, we identified 88 recurrent rearrangement loci across the three breast cancer cell lines (Additional data file 3 [Table S7]).

### Identification of fusion transcripts

**Figure 5:**
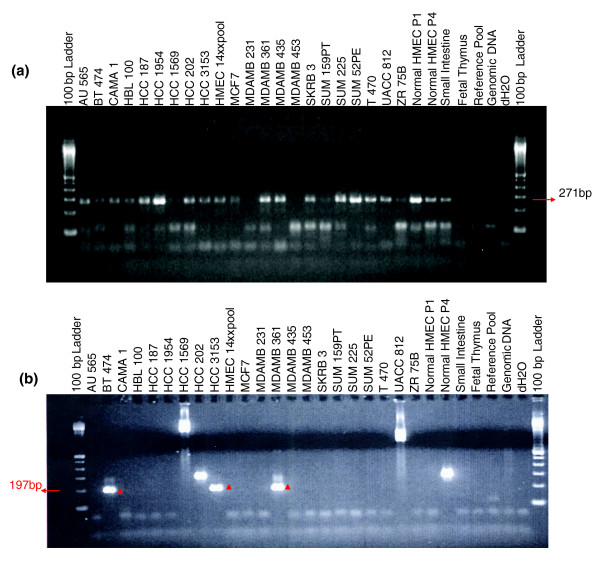
RT-PCR assays of fusion transcripts on a panel of breast cancer cell lines and normal tissues. HMEC-P1 stands for normal human mammary epithelial cells (passage 1), and HMEC-P4 stands for HMEC passage 4 (higher passage). **(a) **RT-PCR reveals expression of DR00074 (*HYDIN *gene fusion) in 16 out of 21 tested breast cancer cell lines, normal cultured human breast epithelial cells, and a wide range of normal human tissues. **(b) **RT-PCR validation of CN272097 a cDNA produced by a complex rearrangement on chromosome 5 fusing the *SLC12A2 *gene and expressed sequence tag (EST) AK090949. The results provide evidence for expression of the fused transcript in 5 out of 21 breast cancer cell lines and in higher passage but not lower passage human mammary epithelial cells (HMECs). Note that MDAMB435 was recently demonstrated to be derivative of the M14 melanoma cell line and not from breast [62], and the absence of the SLC12A2 fusion is this cell line is consistent with its absence in other nonbreast tissues.

Comparison of breakpoints revealed by ESP and putative fusion transcripts identified in public expressed sequence tag (EST) databases provides evidence for expressed gene fusions. In one case, ESP identified two BAC clones spanning an apparent 1q21.1;16q22.2 translocation in MCF7 and a primary breast tumor (MCF7_1-30J11 and 2B421_023-O08, respectively). Both clones were sequenced and found to span identical breakpoints (see Additional data file 3 [Table S8]). An EST clone DR000174 was identified in Genbank that co-localizes with the sequenced breakpoint in BAC clones. This EST fuses a part of exon 6 with an adjoining intron of the *HYDIN *gene to an anonymous gene represented by a cluster of spliced EST sequences. RT-PCR provided clear evidence that the fusion transcript is expressed in 16 out of 21 breast cancer cell lines (Figure 5a and Additional data file 1), normal cultured human breast epithelial cells, and a wide range of normal human tissues. Recently, a 360-kb segmental duplication containing the *HYDIN *locus was identified on chromosome 1q21.1 [28]. This duplication event created the *HYDIN *fusion gene and explains the observed apparent 1q21.1;16q22.2 translocation. To our knowledge this is the first example of a segmental duplication resulting in an expressed fusion gene.

In a second example, a putative fusion transcript (GenBank accession CN272097) and the breakpoint in MCF7 clone 1-97B19 identify a complex rearrangement fusing the SLC12A2 gene and EST AK090949 on chromosome 5. RT-PCR provided evidence for expression of the fused transcript in 5 out of 21 breast cancer cell lines and in higher passage, but not lower passage, human mammary epithelial cells (Figure 5b). In addition, RT-PCR provided clear evidence of alternative splicing of this transcript. Interestingly, we do not detect expression of this fusion transcript in MCF7, possibly because of differences between the location of this breakpoint in MCF7 and the EST. If this fusion is the result of a somatic mutation in breast tumors and not a structural polymorphism, then it will represent the first recurrent fusion transcript reported in breast cancer. Additional studies aimed at analysis of the presence of this transcript in clinical specimens are underway. Thus, paired-end sequencing approaches are useful for the elucidation of genome and transcriptome remodeling in phylogenetics and cancer.

### SNP analysis

**Figure 6:**
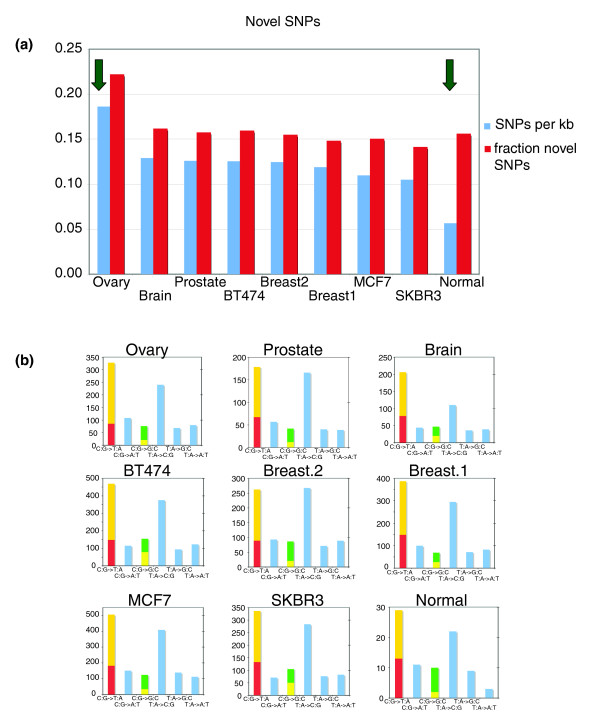
Results of SNP identification in BAC end sequences. **(a) **The number of high quality isolated single nucleotide polymorphisms (SNPs) in uniquely mapped bacterial artificial chromosome (BAC) end sequences expressed per kilobase (blue). Each tumor sample has a significantly higher rate of SNPs compared with the normal library, whereas the ovarian library exhibits a rate significantly higher than the other tumor samples. Also shown is the fraction of SNPs not found in dbSNP124 (red). The ovarian library shows a significantly higher rate of these novel SNPs. **(b) **Mutational spectrum of SNPs for each of the samples. For C:G → T:A transitions and C:G → G:C transversions, the fraction at CpG dinucleotides is indicated in red and yellow, respectively.

The availability of about 89 Mb of sequence from 97,680 mapped BESs made it possible to identify SNPs and candidate somatic mutations. Approximately 62.5% (61,013) of the mapped BESs contained at least one mismatch in the alignment between the BES and the reference genome. From these mismatches, we identified 115,444 candidate SNPs defined as a single base mismatch flanked on both sides by at least one matched base. Many of these mismatches are likely sequencing errors to be expected when examining raw end sequences. Thus, we applied the following filtering criteria to discard low confidence SNPs: the phred score [29] of the SNP, the mean phred score of the five bases centered on the SNP, and the mean phred score of the entire BES containing the SNP all must exceed 30. Approximately 58% of the candidate SNPs were removed by this filtering step, leaving 48,243 SNPs. Of these, 40,659 (84%) are known variants recorded in dbSNP; the probability of this event if our SNP candidates were randomly distributed on the genome, as would be the case if they were largely caused by sequencing errors, is vanishingly small. Thus, our stringent filtering criteria enriched for true SNPs instead of sequencing errors. A total of 7,584 (about 16%) of the valid SNPs are novel (see Additional data file 1 [Table S9]), and 77 of them are recorded in more than one BES (see Additional data file 3 [Table S10]). All of the cancer samples exhibit significantly (*P *< 10^-23^) higher rates of novel SNPs than the normal sample; moreover, the ovarian tumor has a significantly (*P *< 10^-39^) higher rate of SNPs than the other cancer samples (Figure 6). Although some of these novels SNPs are likely to be sequencing errors or rare genetic variants, these cases do not explain the observed biases across samples.

The transition:transversion ratio of these novel candidate SNPs is 1.8, which is lower than the value 1.95 reported for BAC end sequencing of mouse strains [30], comparable to the value 1.85 in coding exons of breast tumors [31], but significantly lower than the value 7.4 in coding exons of colorectal tumors [31]. Moreover, the mutational spectrum of these novel SNPs (see Additional data file 1 [Table S11]) varies across the tumor types, and many of these variations are significant (*P *< 0.00001 by χ^2 ^test). An excess of C:G → T:A transitions over T:A → C:G transitions is observed in all samples except one of the breast tumors, similar to recent reports from exon resequencing studies in tumors [31,32]. However, the asymmetry in the frequency of these two types of transitions is generally less than reported in these studies. Interestingly, the strongest asymmetry is found in our brain sample; this is in agreement with Greenman and coworkers [32], who found the greatest asymmetry in gliomas. Examination of the frequency of variation at dinucleotides (see Additional data file 3 [Table S12]) reveals an excess of C:G → G:C transversions occurring at TpC/GpA dinucleotides, consistent with the report by Greenman and coworkers [32]. The explanation for this bias is not known but is hypothesized to represent a cancer-specific mutational mechanism or environmental exposure.

Thirty-five of the 7,584 novel SNPs were identified in coding regions (see Additional data file 3 [Table S13]). Of these, 24 are nonsynonymous changes that occur in a diverse group of genes, including *IRAK1 *(possibly mutated in breast tumor B421) and *RPS6KB1 *(possibly mutated in BT474), which were previously identified as somatic mutations in breast cancer [33]. Analysis of gene annotations recorded in Gene Ontology with the Database for Annotation, Visualization, and Integrated Discovery (DAVID) tool [34], which corrects for differences in the sizes of annotated gene families, identified six genes classified as 'transition metal ion binding' (*P *= 0.07), including the zinc-binding proteins encoded by *ZNF217*, *ZNF160*, *ZNF354C*, *ZDHHC4*, and *ANKMY1*. Interestingly, the SNP in *ZDHHC4 *occurs in the zinc finger domain, as defined in UniProt. Examination of SNPs in amplified regions in MCF7, BT474, and SKBR3 did not suggest any correlation between SNP rate and amplification; some amplicons harbor a high number of sequence variants, whereas others have relatively few (see Additional data file 3 [Table S14]).

We resequenced 17 candidate SNPs found in the breast cancer cell lines (see Additional data file 3 [Table S15]) and confirmed 11 out of 17 (64.7%), a success rate very similar to the 68% reported in large-scale resequencing of exons [31]. Of the six remaining cases, four were sequencing failures, whereas two contained double signals in the ABI electrophoregrams at the SNP site, with the reference peak being the dominant one. Thus, it is possible that these SNPs are heterogeneous in the cell lines. Therefore, only 2 out of 17 candidate SNPs (11.8%) were contradicted by resequencing. Because 2 of the 11 validated SNPs, plus two that were not validated, were also found in a more recent update of dbSNP (128), we checked all 7,584 novel SNPs against dbSNP Build 128. We found that 1,698 (22%) were present, providing further evidence that our SNP filtering criteria are enriching for true sequence variants rather than sequencing artifacts.

## Discussion

The importance ascribed to different types of genome aberrations in cancer is frequently directly coupled to the technology available to measure them; classic cytogenetics demonstrated the functional significance of translocations in tumors with simple karyotypes, whereas loss of heterozygosity, CGH, and array-CGH studies have led to an explosion of interest in recurrent copy-number aberrations. More recently, targeted [32,35] and whole genome exon resequencing [31] has demonstrated the importance of coding mutations. The Cancer Genome Atlas project [36] promises to increase drastically the number of known coding somatic mutations. However, it is likely that structural rearrangements in tumor genomes are as important to tumor biology and the development of biomarkers and therapeutics as are coding point mutations [37,38]. We have demonstrated that ESP provides direct access to the structural complexity of tumor genomes by identifying and cloning all classes of structural rearrangements, including fusion genes and their transcripts. ESP also proved to be a powerful tool for analysis of structural polymorphism present in the normal human genome [39,40]. Moreover, identification of the *HYDIN *gene fusion by ESP reveals that duplicon-mediated genome rearrangements can result in expression of structurally novel genes. Using this approach, it is also possible to survey the spectrum of mutations and/or SNPs present in a tumor genome in an unbiased manner.

Many of the recurrent breakpoints that we identified arise from micro-rearrangements of less than 2 Mb (Figure 4). Although some of these rearrangements are likely to be novel structural polymorphisms, micro-rearrangements have also been observed in evolution [41,42] and in some tumors [43]. Because micro-rearrangements are largely invisible to cytogenetic techniques, the collection of the breakpoints reported in this paper provides an excellent resource for future studies of the mechanisms, prevalence, and consequences of these micro-rearrangements in tumorigenesis.

Sequencing BAC clones identified by ESP was performed to localize and validate about 90 breakpoints in this and in a previous study [7]. To our knowledge, this is currently the largest collection of sequenced rearrangement breakpoints in cancer. Importantly, this collection can be easily extended as needed, because ESP also created the largest collection to date of hundreds of sequence-ready breakpoint-spanning BAC clones. Most breakpoint-spanning BAC clones, including all BAC clones sequenced from primary tumors, contain single breakpoints. However, in the three cell lines, 17 clones containing multiple breakpoints were identified and confirmed by PCR. These observations were supported by DNA fingerprinting (Marra M, personal communication) [21]. The observed differences between the primary tumors and cell lines may be due to genomic heterogeneity (and consequently lower sequence coverage) of tumor samples, differences in tumor type and/or stage, or intrinsic differences in genomic organization between cell lines and primary tumors. It will be informative to perform ESP on primary breast tumors with copy-number profiles very similar to those of the cell lines studied here [10,11] and to establish the degree of the structural similarity of the samples with similar copy-number and expression profiles.

Our analyses of breakpoint junction sequences revealed that the overwhelming majority of identified rearrangements (96%) are consistent with aberrant NHEJ repair. This observation is consistent with the previously reported predominant role of nonhomologous recombination in generation of pathologic translocations [44] and in frequent rearrangements at chromosomal ends [45]. Although there are reports of associations between locations of cancer breakpoints and evolutionary breakpoints [46], ESP data did not reveal a significant association in our samples (data not shown).

We used sequenced breakpoints to refine the mapping of amplicon structures in MCF7 using PCR in seven independent BES clusters. This process identified breakpoint heterogeneity in five clusters (Figure 2 and Additional data file 2 [Figure S3]). One explanation for this phenomenon is variability in the location of breakpoints in multiple fusions of the same loci, analogous to the variability of breakpoints in fusion genes in hematopoietic malignancies. Alternatively, the heterogeneity might reflect early events present in a minority of cells in the population. To our knowledge, this is the first example of structural heterogeneity observed on a molecular level in tumor genomes.

Analysis of SNPs in BAC end sequences identified elevated rates of SNPs in each tumor sample compared with the normal sample, with the ovarian tumor exhibiting a rate significantly above the other samples. Although the ability to distinguish somatic mutations from sequencing errors or germline mutations is limited in the present study, there is no reason to suspect that these confounding factors vary enough between samples to explain the observed differences. The mutational spectra of SNPs in these samples share some features with those from exon resequencing studies [31,32], but there are also many differences. These differences might be due to different mutational biases in coding regions, but further study is needed to support this hypothesis. Given that the BES arise from a genome-wide survey, it is not surprising that we identify few candidate mutations in coding regions. However, it is intriguing that even the relatively small numbers of putative mutations are enriched for zinc finger genes, including the known breast cancer oncogene *ZNF217 *[27,47,48].

Using ESP it is possible to reconstruct tumor genome structure and evolution [4-7]. ESP data from the three breast cancer cell lines identify clones that fuse noncontiguous amplified loci, possibly suggesting functional coupling of co-amplified genes. The discovery of recurrent breakpoints and regularly spaced breakpoints in the cell-line data could be a molecular signature of breakage/fusion/bridge (B/F/B) cycles [7]. In some cases, ESP data suggest a specific temporal progression in which amplification follows translocations or deletions. For example, a cluster of 19 clones span a 17;20 translocation in MCF7. This coverage is highly unlikely (*P *< 10^-20^) for a nonamplified locus, and PCR mapping confirmed identical breakpoints in these clones. The most parsimonious explanation is that the translocation preceded the amplification. In a second example, a cluster of six BT474 clones spans a deletion. Once again the simplest explanation is that the deletion preceded amplification of the surrounding locus, because a cluster of size six clones is highly unlikely (*P *≈ 10^-5^) in a nonamplified locus. Interestingly, this deletion may truncate the *THRA1 *gene, as reported by Futreal and coworkers [25], and fuse it to the *SCAP1 *gene. Amplification of a breakpoint might occur because the fused genomic region encodes a fusion gene that confers a selective growth advantage. Alternatively, amplification might be a random byproduct of genomic instability near the rearrangement breakpoint. Regardless, the breakpoint information is valuable for determining the temporal evolution of tumor genome organization.

The identification of *TMPRSS2 *translocations in about 50% of prostate tumors [3] underscores the significance of structural rearrangements in solid tumors. Although our prostate sample does not contain the *TMPRSS2 *translocation (Rubin M, personal communication), ESP mapping and breakpoint sequencing provide numerous examples of possible gene fusions, including the previously published *BCAS4/3 *fusion in MCF7. Moreover, integration of public EST data with ESP data demonstrates that this approach can identify fusion transcripts *en masse*. We identified a fusion transcript that results from an evolutionarily recent rearrangement of the normal genome and obtained evidence for the first recurrent fusion transcript in breast cancer. In this study the clonal coverage of tumor genomes ranged from only 0.15-fold to 0.7-fold redundancy. It is probable that many additional gene fusions will be identified upon deeper paired end analysis of both normal and tumor genomes and transcriptomes.

The extension of ESP to multiple tumor types demonstrates that its application is not restricted to specific tumor types and that ESP functions well even with small tumor specimens. This is important because advances in diagnostics have resulted in a reduction in the average volume of many surgically excised tumors. For example, the average size of breast tumors excised before 1985 was 25 mm, whereas after 1985 it decreased to 21 mm [49], a 1.6-fold decrease in the volume of excised breast tumors. Moreover, tumor heterogeneity and normal cell admixture necessitates dissection further reducing subsequent yields of tumor cell DNA. Finally, clinically annotated tumor specimens are an extremely valuable resource and should be used as sparingly as possible. Therefore, it is significant that we were able to construct a tumor BAC library from less than 20 mg of a frozen and partially necrotic tumor (B421).

DNA yields from the tumors suggest that libraries comprised of 200,000 to 400,000 clones are possible, meaning that the genomes of these tumors can be immortalized and made widely available. This study demonstrates the utility of ESP for whole genome screening of SNPs/mutations. The immortalization of the tumor genome in a clone library is important, because some studies report underestimation of the mutation load because of heterogeneity in tumors [50], and overcoming this problem will require either development of the novel software or implementation of the novel sequencing technologies, allowing analysis of single DNA molecules [51]. Because clone libraries can be duplicated and their DNA pooled, it becomes feasible to perform large exon resequencing projects on small tumors, such as those of the breast and prostate. In addition, because BAC clones contain DNA from a single tumor cell, identification of rare SNPs/mutations in heterogeneous tumors is theoretically possible in a manner analogous to the identification of breakpoint heterogeneity in tumor amplicons reported here. Finally, the ability to rapidly identify sequence variants in DNA pools and to then recover the physical clone means that studies aimed at determining the biologic relevance of the variants are possible using established *in vivo *and *in vitro *systems.

ESP is less impeded by tumor heterogeneity or contamination by normal cells than is aCGH, because each end sequenced clone originates from a single DNA molecule from a single cell. Deep sequencing of many clones allows one to overcome normal tissue admixture and enables direct measurements of heterogeneity and detection of rare events. Eventually it will be possible to apply techniques from metagenomics [52] to study the heterogeneous pool of cells that are present in early stage tumors, with the goal of identifying the earliest informative biomarkers and therapeutic targets. At present, the relatively high cost of ESP limits its application to a small number of tumors, but advances in massively parallel sequencing technologies capable of paired-end sequencing (for review [9]) will permit large-scale ESP studies at a fraction of the current cost. However, much of the cost savings realized by the current crop of next generation sequencing technologies result from skipping the immortalization of the tumor genome as a clone library. Such cloning enables further sequencing of breakpoints and evaluation of their functional significance via *in vitro *and *in vivo *assays [7]. Combining ESP with such assays will enable tumor progression studies aimed at identification of events linked to initiation, progression, and metastasis. Thus, although the selection of a particular implementation of ESP will be driven by the cost/benefit analysis for the specific goals of the project, paired end sequencing approaches promise to revolutionize our understanding of the complex organization of the genomes of solid tumors.

## Materials and methods

### BAC library construction

Breast cancer cell lines were obtained from University of California, San Francisco (UCSF) cell culture facility. Clinical tumor specimens were obtained from the Bay Area Breast Oncology Program (breast tumors), rapid autopsy program at the University of Michigan [53], and the University of Texas MD Anderson Cancer Center SPORE in ovarian cancer (ovarian).

Library preparation was carried out as described previously [7] (see detailed protocol on the internet [54]). Briefly, fresh frozen tissue (0.1 to 0.15 g) was slowly thawed on ice, ground, and re-suspended in 0.6 ml of 1× phosphate-buffered saline (pH 7.0). The suspension was pre-warmed to 42°C in water bath and mixed with an equal amount of a warm 1.5% solution of low melting-point agarose. The partial restriction was carried out for 1 hour on ice, followed with incubation for 10 minutes at 37°C and stopped by addition of 0.1 volume of 0.5 mol/l EDTA. Additional processing associated with isolation of high molecular weight DNA, construction of BAC libraries, and end sequencing of BAC clones was carried out as previously reported [7].

### ESP data analysis

We employed a two-step procedure that involved first mapping the BES data onto the human genome sequence (National Center for Biotechnology Information [NCBI] build 35, May 2004), and then filtering the mapping results. The mapping step is accomplished using BLAST-like alignment tool (BLAT) [55]. A location is assigned if at least 50 bp of a BES aligned to the reference genome sequence with at least 97% identity. If the BES hit multiple locations in the genome, then the position of the longest hit with highest identity was chosen and the BES was designated as being 'ambiguously mapped' and excluded from further analysis. Finally, BES mapping to known segmental duplications, as defined by the SegmentalDups track of the UCSC Genome Browser, were removed. Only clones corresponding to unique BES pairs were retained. BES mappings are available as a custom track for the UCSC Genome Browser on the internet [56].

BES pairs with BES mapping to the same chromosome and having opposite convergent orientations (for instance, a pair of the form [(chrom1, loc1, strand1), (chrom2, loc2, strand2)] with chrom1 = chrom2, loc1 < loc2, strand1 = '+', and strand2 = '-') were identified. The distribution of distances between mapped ends (loc2 to loc1) was used to define the length distribution of the BAC libraries. BES pairs with ends on the same chromosome and having convergent orientations on opposite strands and distances in the 99.5% quantile of this distribution were classified as valid. Other BES pairs were classified as invalid and thus candidate rearrangements in the tumor. Note that the distance criterion was very permissive and might misclassify clones harboring small indels as valid. Overlapping valid pairs were combined into 'contigs', whereas invalid pairs were clustered into sets according to whether their locations were close enough to be explained by a single rearrangement event [4-7]. Invalid pairs (or clusters) were classified as potential indels, inversions, or translocations, according to the location and orientation of their ends (see Additional data file 1 [Table S1]).

Custom software was used to visualize the mapping results, as described by Volik and cowokers [6]. A plot of BES density generated a copy number profile for the entire tumor genome, because the overall number of BESs per given genomic interval is roughly proportional to copy number.

### Known structural variants

Locations of previously reported structural variants were downloaded from the Database of Genomic Variants [23,57]. Clusters of invalid BES pairs were labeled as 'explained' by the known structural variant if the locations of the variant overlapped the locations of an end sequence pair in the cluster, and the type of variant was consistent with the orientations of the mapped end sequences in the clusters. That is, pairs with convergent orientation are consistent with insertions and deletions (copy number variants), whereas pairs with the same orientation are consistent with inversions. We did not require precise overlap between the breakpoints of the invalid BES pairs and the breakpoints of the structural variants because both types of breakpoints were only approximately known. Note that multiple structural variants might 'explain' a cluster because the structural variants in the database were merged from different experimental sources and have some redundancy [58].

### BAC sequencing

BAC DNA was purified from 250 ml overnight culture using the Qiagen columns (Qiagen, Hilden, Germany). Approximately 2 μg of BAC DNA was mechanically sheared using the HydroShear (Genomic Solutions Inc., Ann Arbor, MI, USA), end-repaired with the Klenow enzyme and T4 DNA polymerase, size selected for 3 ± 0.5 kb fragments on agarose gels, and cloned into a pUC19 vector. Individually picked subclones were grown on 96-well plates overnight in LB plus 200 μg/ml ampicilin and 10% glycerol. Plasmid DNA was prepared from the arrayed cells using the TempliPhi kit (GE/Amersham, Chalfont St. Giles, UK), in accordance with the manufacturer's protocol. Three-kilobase subclones were end sequenced using BigDye terminators (Applied Biosystems, Foster City, CA, USA) and capillary sequencers. The quality of the sequence reads were determined by Phred score [29], and only sequences greater than Q20 were included in the analysis.

### Analysis of rearrangements breakpoint junctions

Breakpoint junction sequences were aligned to the Human Genome Assembly (NCBI build 35, May 2004) using BLAT [55], and the alignments were analyzed for the precise position of the breakpoint and presence of microhomologies. Breakpoint sequences were also analyzed for their repeat content using the RepeatMasker program and for their overlap with known copy number polymorphic regions using the Structural Variation track of the Genome Browser. The mechanism of each rearrangement was deduced from the alignment of the breakpoint junction sequence to the native sequences of the two regions participating in the rearrangement, and the number of total DSBs calculated as previously described [45].

### SNP analysis

Out of the approximately 70,000 clones sequenced for this and previous studies, we selected the 97,860 BESs that mapped to unique loci on the hg17 reference genome with a minimum BLAT identity score of 97%. The mean phred score [29] of these BESs is 51. A total of 61,013 of the selected BESs contained at least one mismatch. Runs of multiple contiguous mismatches and indels were not considered when defining a SNP. We identified 115,444 candidate SNPs, which we defined as a single base mismatch flanked on both sides by at least one matched base. A total of 67,201 (58.21%) of these candidate SNPs were attributed to possible sequencing errors, because the phred score of the SNP, or the mean phred score of the five bases centered on the SNP, or the mean phred score of the entire BES was below 30. Candidate SNPs were not considered tumor specific if their location and nucleotide change matched a known SNP in dbSNP build 124. Coding SNPs were identified as those than lie in exons annotated from the Known Genes track of the UCSC Genome Browser. The observed rates of SNPs of each type of nucleotide substitution were compared across different samples using the χ^2 ^test. Enrichment of Gene Ontology terms for the genes containing candidate SNPs was computed with the DAVID tool [34], which computes *P *values for enrichment correcting for the size of the gene sets in each term. We used the LiftOver tool from the UCSC Genome Browser [59] to identify the locations of each novel SNP in the latest build (build 36) of the human reference genome and examined whether these SNPs were present in dbSNP build 128 using the snp128 table. The validation of candidate SNPs/mutations was performed by direct genomic sequencing of the DNA amplified from the cell line used for ESP.

### RT-PCR

RT-PCR experiments were carried out as described by Zardo and coworkers [60]. Primer sequences and conditions are presented below. We employed a nested PCR strategy to increase specificity and sensitivity of our assay. All PCR reactions were carried out in 25 μl reaction volumes using following program: initial denaturation of DNA, 4 minutes at 94°C; 30 to 35 cycles of 15 seconds at 94°C; 30 seconds at 60°C; and 45 seconds at 72°C. We have used about 100 ng of cDNA for the first reaction with outer primers, and 1 μl of the resulting PCR reaction for the second round using inner primers. The following primers were used. For DR00074 we used AGGAAAAGGCCTTGAAGCTC and TGCTGTATTTGACAGGACAAGTG (outer primers), and GAGGACATGCTCCTACCTGTG and TGCTGTATTTGACAGGACAAGTG (inner primers). For CN272097 we used CCAACGTGAGCTTCCAGAAC and ACAGAAACGCCTCTTCTCATTTAG (outer primers), and TATTATGATACCCACACCAACACC and CTCCTGTTCGTGTCAGCAATAC (inner primers). The specificity of PCR reactions were validated by sequencing at UCSF Genome Analysis Core.

### Spectral karyotyping and FISH analysis

Cells lines were shipped to Dr. Padilla-Nash. When cell lines reached 70% confluence, cells were treated with colcemid (Roche, Indianapolis, IN, USA) for 1 hour to arrest the cells in mitosis. Metaphase chromosome suspensions were prepared first by treating cells with a hypotonic solution (0.075 mol/l KCl); next, the cells were fixed using methanol:acetic acid (3:1, vol/vol) and dropped onto slides in a humidity controlled chamber. The slides were aged at 37°C for approximately 1 week. Chromosome preparations were hybridized with either FISH probes or spectral karyotyping (SKY) probes for 72 hours. The protocols for preparation of FISH/SKY probes, slide pre-treatment, slide denaturation, detection, and imaging have been described previously and are available on the internet [61]. Ten to fifteen metaphase spreads were analyzed per sample and scored for the following: chromosome number (ploidy), numerical aberrations, and structural aberrations. Spectrum-based classification and analysis of the fluorescent images (SKY) was achieved using SkyView™ software (Applied Spectral Imaging, Carlsbad, CA, USA). The karyotypes of every metaphase spread from all groups were characterized using the human chromosome nomenclature rules adopted in the 2005 International System for Human Cytogenetics Nomenclature.

## Abbreviations

aCGH, array comparative genomic hybridization; BAC, bacterial artificial chromosome; BES, BAC end sequence; BLAT, BLAST-like alignment tool; DAVID, Database for Annotation, Visualization, and Integrated Discovery; DSB, double strand break; ESP, end sequencing profiling; EST, expressed sequence tag; FISH, fluorescence *in situ* hybridization; kb, kilobase; Mb, megabases; NCBI, National Center for Biotechnology Information; NHEJ, nonhomologous end joining; PCR, polymerase chain reaction; RT, reverse transcription; SKY, spectral karyotyping; SNP, single nucleotide polymorphism; UCSF, University of California, San Francisco.

## Authors' contributions

BJR analyzed the data including identification and analysis of sequence variants, clustering of the identified breakpoints, and comparison of ESP and aCGH data. SV and CC developed the ESP methodology and BES mapping algorithms, analyzed the data and coordinated the clinical samples, sequencing, and experimental validation of ESP results. PY and CW integrated ESP and public EST data, and identified fusion transcripts. EL and BT performed analysis of sequenced breakpoints and contributed to paper writing. FW selected and managed the breast clinical specimens and developed the FISH methods of breakpoint validation. JC, KJP, and GBM managed and selected the brain, prostate, ovary tumor samples, respectively. PP, KB, YK, G-QH, and SS performed experimental validation. AB, RB, and SJA performed analysis of fusion genes and sequence variants. JWG and J-FC sequenced BAC clones. QT, PdJ, and MN constructed BAC libraries. HP-N and TR performed FISH validation and experimental validation of ESP breakpoints. BJR, SV, and CC wrote the paper. All authors read and approved the final manuscript.

## Additional data files

The following additional data are available with the online version of this paper. Additional data file 1 contains supplemental text and tables, including a description of all supplemental tables. Additional data file 2 contains three supplemental figures. Additional data file 3 contains supplemental tables.

## Supplementary Material

Additional data file 1 Supplemental text and tables, including a description of all supplemental tables. Click here for file

Additional data file 2 Three supplemental figures. Click here for file

Additional data file 3 Supplemental tables. Click here for file
