# Supplementary material for: A sequence-based survey of the complex structural organization of tumor genomes
Source: Genome Biol. 2008 Mar 25;9(3):R59. doi: 10.1186/gb-2008-9-3-r59 (PMC2397511; doi:10.1186/gb-2008-9-3-r59)
Supplement: Additional data file 1 — Supplemental text and tables, including a description of all supplemental tables. [file gb-2008-9-3-r59-S1.pdf]

A sequence-based survey of the complex structural organization of tumor genomes

Raphael, et. al

Supplemental Information

Supplemental Text

BAC Sequencing

Breakpoints in six clones could not be identified due to repetitive elements and genome assembly problems. In the case of the IGBR10_H22 brain clone no evidence of rearrangement was detected in plasmid reads. Subsequent analysis demonstrated that the original identification of this clone as rearrangement-spanning was due to errors in the older version of human genome assembly. Clones IGBR03_C11 from the brain library, 2B421_009_P13 2B421_001_H08 from B421 the primary breast library and MCF7_1-151N7 from MCF7 library mapped to regions dense in high-copy repeats, and assembly proved to be impossible. We have not been able to sequence across the breakpoint in prostate clone PM1_009_L12 for unknown reasons.

Comparison to Known Cell Line Data

ESP recapitulates known features of these cell lines, including amplification of 20q13 in MCF7 and BT-474, the BCAS4/3 fusion in MCF7 [1], and amplification of 17q including the *HER2/ERBB2* oncogene in MCF-7. The largest BES cluster in BT474 spanned an apparent 17q12-q21.3 deletion, which was previously reported [2]. This study also reported a fusion involving the THRA1 gene, which is near the breakpoint region of our cluster. In comparison, earlier ESP analysis of MCF-7 [3] also showed amplification of chromosomes 17q at different loci. Moreover, we found MCF-7 to have a significantly greater fraction of rearranged clones, consistent the fact that the MCF-7 cell line is derived from a metastatic tumor while BT-474 was derived from a primary tumor. SKBR3 data reveals significant amplification and rearrangement of chromosome 8q, consistent with earlier studies including cDNA based copy number analysis which revealed three distinct amplicon peaks on 8q [4].

Fusion Transcript Identification

Putative fusion transcripts identified in ESP and EST data were assayed in the breast cancer cell lines: AU565, BT474, CAMA1, HBL100, HCC187, HCC 1954, HCC 1569,

HCC202, HCC3153, MCF7, MDAMB231,MDAMB 361, MDAMB435, MDAMB 453, SKRB3, SUM 159PT, SUM 225, SUM52PE, T470, UACC812, ZR75B.

References

1. Barlund M, Monni O, Weaver JD, Kauraniemi P, Sauter G, Heiskanen M, Kallioniemi OP, Kallioniemi A: **Cloning of BCAS3 (17q23) and BCAS4 (20q13) genes that undergo amplification, overexpression, and fusion in breast cancer**. *Genes Chromosomes Cancer* 2002, **35**(4):311-317.
2. Futreal PA, Cochran C, Marks JR, Iglehart JD, Zimmerman W, Barrett JC, Wiseman RW: **Mutation analysis of the THRA1 gene in breast cancer: deletion/fusion of the gene to a novel sequence on 17q in the BT474 cell line**. *Cancer Res* 1994, **54**(7):1791-1794.
3. Volik S, Raphael BJ, Huang G, Stratton MR, Bignel G, Murnane J, Brebner JH, Bajsarowicz K, Paris PL, Tao Q *et al*: **Decoding the fine-scale structure of a breast cancer genome and transcriptome**. *Genome research* 2006, **16**(3):394-404.
4. Pollack JR, Sorlie T, Perou CM, Rees CA, Jeffrey SS, Lonning PE, Tibshirani R, Botstein D, Borresen-Dale AL, Brown PO: **Microarray analysis reveals a major direct role of DNA copy number alteration in the transcriptional program of human breast tumors**. *Proceedings of the National Academy of Sciences of the United States of America* 2002, **99**(20):12963-12968.

Supplemental Tables

Table 1: Number of invalid BES pairs and clusters suggestive of particular rearrangements. The p-value is the probability that the fraction of invalid pairs is the same as observed in the normal library, using a sample proportion test with pooled variance.

	MCF7	BT474	SKBR3	Breast	Breast.2	Prostate	Ovary	Brain	Normal
Invalid	491	186	187	164	113	96	87	67	8
Num clusters	36	26	24	2	7	2	2	0	0
Pairs in clusters	164	61	64	4	24	4	4	0	0
Pairs translocations	261	36	31	73	21	51	11	28	2
p-value	**0.001**	0.337	0.372	**0.044**	0.445	**0.012**	0.411	**0.086**	NA
Clusters translocations	19	5	5	0	1	1	0	0	0
Singletons translocations	169	25	21	73	19	49	11	28	2
Pairs inversions	81	56	61	19	33	7	13	4	2
p-value	0.160	0.144	**0.093**	0.398	0.219	0.262	0.497	0.117	NA
Clusters inversions	9	10	9	0	4	0	0	0	0
Singletons inversions	42	33	34	19	22	7	13	4	2
Pairs deletions	110	74	68	64	52	32	36	30	4
p-value	0.272	0.258	0.259	0.260	0.269	0.271	0.270	0.263	NA
Clusters deletions	4	8	4	1	2	1	0	0	0
Singletons deletions	99	53	56	62	41	30	36	30	4
Pairs in cent/tel	18	13	9	17	15	6	3	4	0
Clusters in cent/tel	0	4	0	0	2	0	0	0	0

Table 2: Results of BAC clone draft sequencing.
(See Additional data file 3.)

Table 3: Analysis of breakpoint junctions.
(See Additional data file 3.)

Table 4: PCR validation of clusters.
(See Additional data file 3.)

Table 5: Known structural variants overlapping recurrent clusters found in ESP.
(See Additional data file 3.)

Table 6: Recurrent clusters indicating a pericentric inversion on chromosome 11, a deletion on chromosome 10 in a breast tumor and cell line, and a deletion in two breast cancer cell lines.

Cluster 1	Cluster 2	Cluster 3
((11, 51023803, -), (11, 54672066, -), 2B421_020_N10)	((10, 47542232, +), (10, 51840247, -), CHORI514_8_F16)	((17, 53838744, +), (17, 56182504, -), CHORI518_021_O23)
((11, 51027332, -), (11, 54698532, -), CHORI514_1_J16))	((10, 47556097, +), (10, 51856818, -), CHORI518_007_F02)	((17, 54420862, +), (17, 55271743, -), MCF7_1-151N7)
((11, 51056054, -), (11, 54657483, -), CHORI514_7_B06)	((10, 47556101, +), (10, 51823897, -), CHORI518_013_M19)	((17, 54424526, +), (17, 55279688, -), MCF7_1-13G5)
((11, 51088628, -), (11, 54575927, -), CHORI518_007_N06)		((17, 54447887, +), (17, 55269584, -), MCF7_1-52j15)
((11, 51140215, -), (11, 54471902, -), MCF7_1-30c02)		
((11, 51151679, -), (11, 54503063, -), CHORI520_R1_26_K13)		

Table 7: Recurrent rearrangement loci from MCF7, BT474, and SKBR3 defined as BES from different samples mapped within 263kb.
(See Additional data file 3.)

Table 8: The locations of clones and ESTs indicating shared fusion transcripts. The indicated tissue source for each EST is from the GenBank record. The origin of the adenocarcinoma library for BU944045 is unknown, but MCF7 is the most likely source as the transcript corresponds to MCF7-specific BCAS4/3 fusion transcript.
(See Additional data file 3.)

Table 9: Results of mutation identification in BAC end sequences (BES). A valid SNP is defined as a single base pair change where the minimum of: the average PHRED score for the BES, the PHRED scores at the base, in the PHRED scores in the 5 bases surrounding the mutated base, exceeds 30. Novel SNPs are those not present in dbSNP124, and p-values indicate probabilities of obtaining the given number (fraction) of novel SNPs compared to the number (fraction) in the normal sample.

Sample	Library Name	Nucleotides Sequenced	Valid SNPs	Novel SNPs	Novel SNPs/Mb	Pval (novel)	Fraction Novel	p-value (fraction)
**Ovary**	**CHORI510**	**4,862,337**	**4,074**	**900**	**185.1**	**1.6E-29**	**22.1%**	**2.1E-04**
Brain	IGBR	3,765,830	2,998	482	128.0	4.5E-24	16.1%	3.7E-01
Prostate	PM1	4,182,264	3,336	522	124.8	2.0E-25	15.6%	4.6E-01
BT474	CHORI518	10,704,340	8,388	1,330	124.2	1.6E-72	15.9%	4.1E-01
Breast_2	CHORI514	7,033,022	5,641	868	123.4	1.1E-44	15.4%	4.7E-01
Breast_1	2B421	8,531,797	6,826	1,005	117.8	3.2E-48	14.7%	3.1E-01
MCF7	MCF7	13,255,081	9,644	1,440	108.6	7.7E-60	14.9%	3.6E-01
SKBR3	CHORI520	9,173,728	6,794	953	103.9	1.4E-34	14.0%	1.7E-01
**Normal**	**K0241**	**1,515,195**	**542**	**84**	**55.4**		**15.5%**	
Total/Mean		63,023,594	48,243	7,584	120.3		15.7%	

Table 10: SNPs identified in more than one BAC end sequence.
(See Additional data file 3.)

Table 11: Frequencies of novel SNPs in each sample, with percent of total in parentheses.

	MCF7	BT474	SKBR3	Breast	Breast.2	Prostate	Ovary	Brain
Total	1440	1330	953	1005	868	522	900	482
C:G-->T:A	505 (35.1)	469 (35.3)	336 (35.3)	387 (38.5)	262 (30.2)	178 (34.1)	328 (36.4)	206 (42.7)
C:G-->G:C	125 (8.68)	155 (11.7)	105 (11.0)	69 (6.87)	86 (9.91)	42 (8.05)	76 (8.44)	47 (9.75)
C:G-->A:T	151 (10.5)	115 (8.65)	71 (7.5)	100 (9.95)	93 (10.7)	57 (10.9)	108 (12.0)	44 (9.13)
T:A-->C:G	408 (28.3)	375 (28.2)	283 (30.0)	295 (29.4)	267 (30.8)	166 (31.8)	240 (26.7)	110 (22.8)
T:A-->G:C	139 (9.65)	94 (7.07)	76 (8.0)	71 (7.06)	71 (8.18)	40 (7.66)	68 (7.56)	36 (7.47)
T:A-->A:T	112 (7.78)	122 (9.17)	82 (8.6)	83 (8.26)	89 (10.3)	39 (7.47)	80 (8.89)	39 (8.09)

Table 12: Frequency of novel SNPs at specific dinucleotides in each sample.
(See Additional data file 3.)

Table 13: Novel candidate mutations in BAC end sequences that map to known genes in the UCSC Genome Browser. Non-synonymous changes are indicated in bold.
(See additional data file 3.)

Table 14: Frequency of SNPs in amplicons in MCF7, BT474, and SKBR3.
(See additional data file 3.)

Table 15: Results of PCR validation of novel SNPs.
(See additional data file 3.)
