# Supplementary material for: A sequence-based survey of the complex structural organization of tumor genomes
Source: Genome Biol. 2008 Mar 25;9(3):R59. doi: 10.1186/gb-2008-9-3-r59 (PMC2397511; doi:10.1186/gb-2008-9-3-r59)
Supplement: Additional data file 2 — Three supplemental figures. [file gb-2008-9-3-r59-S2.pdf]

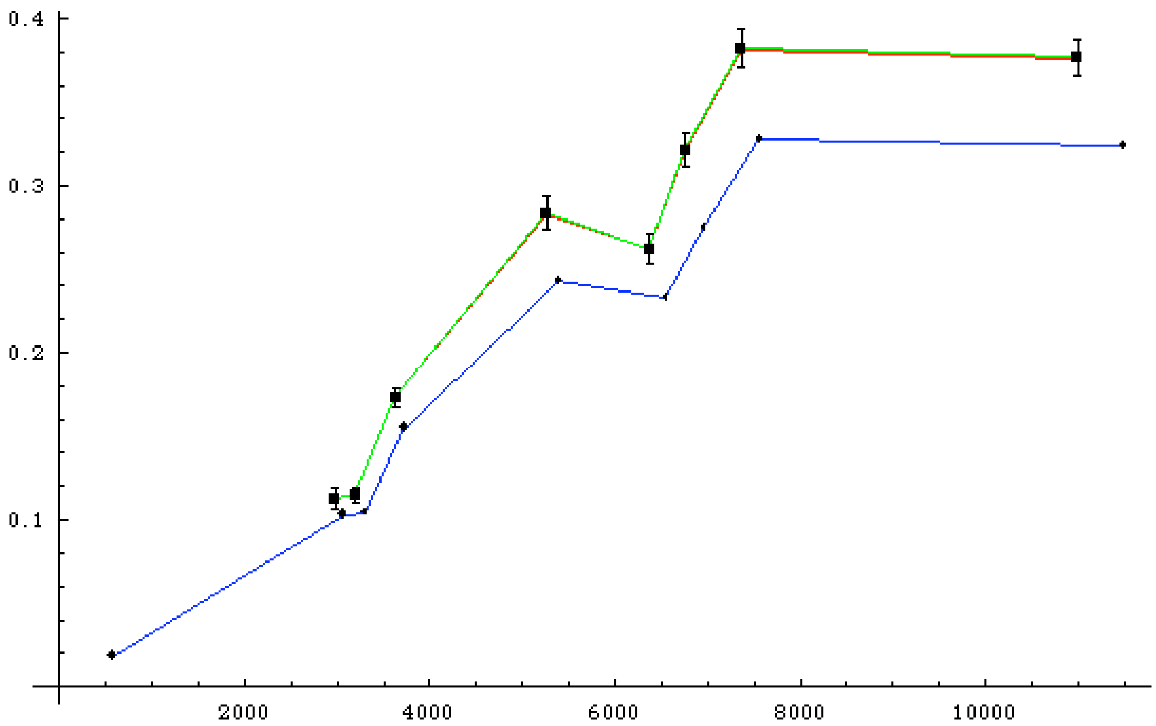

Figure S1: (Blue) Each data point indicates the corresponding clonal coverage of the human genome for ESP data from normal, brain, ovary, prostate, breast (2B421), breast (CHORI514), SKBR3, BT474, and MCF7 samples. (Red) Each data point indicates the clonal coverage predicted by Lander-Waterman statistics using the mean BAC size for each library. (Green) Each data point indicates the mean clonal coverage obtained by resampling random BACs from empirical length distribution of each library. Error bars represent ten standard deviations from the mean.

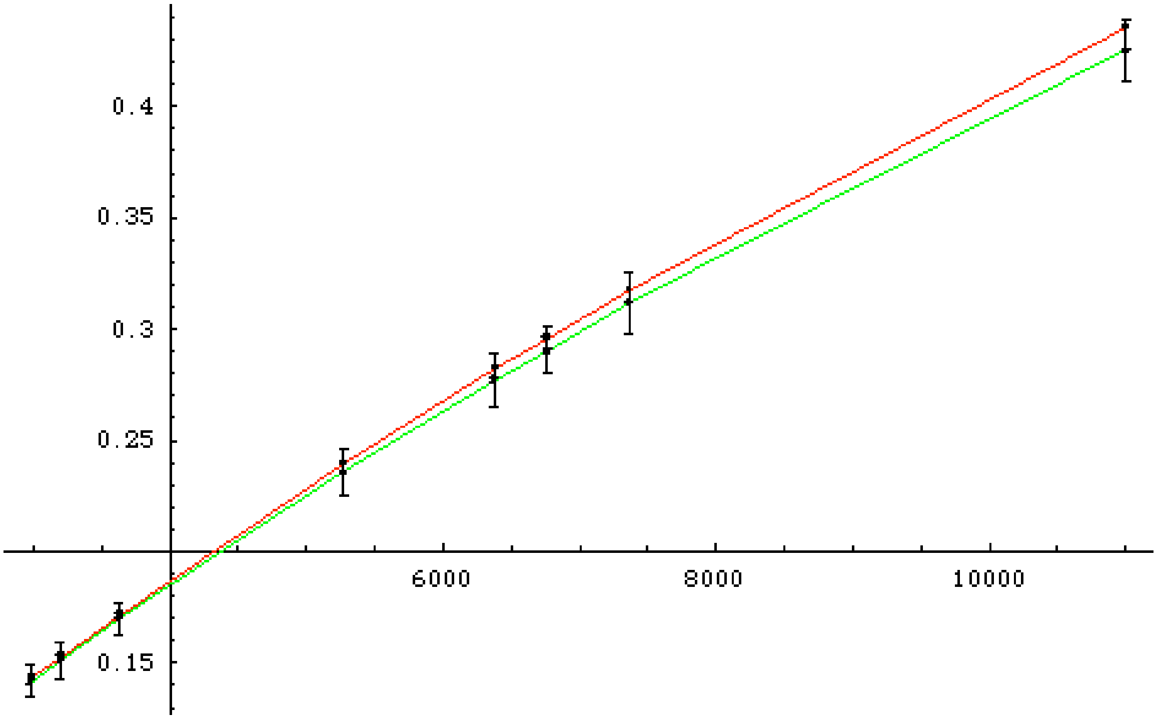

Figure S2: Comparison of coverage of the human genome predicted using Lander Waterman statistics (red) and empirical coverage from resampling of mapped BACs from the "BAC end pairs" track of the UCSC Genome Browser (green). Error bars represent ten standard deviations from the mean. Empirical coverage is consistently lower than predicted but within 10 standard deviations in all cases, in contrast to the lower coverage observed for the tumor and cell line libraries in Figure S1.

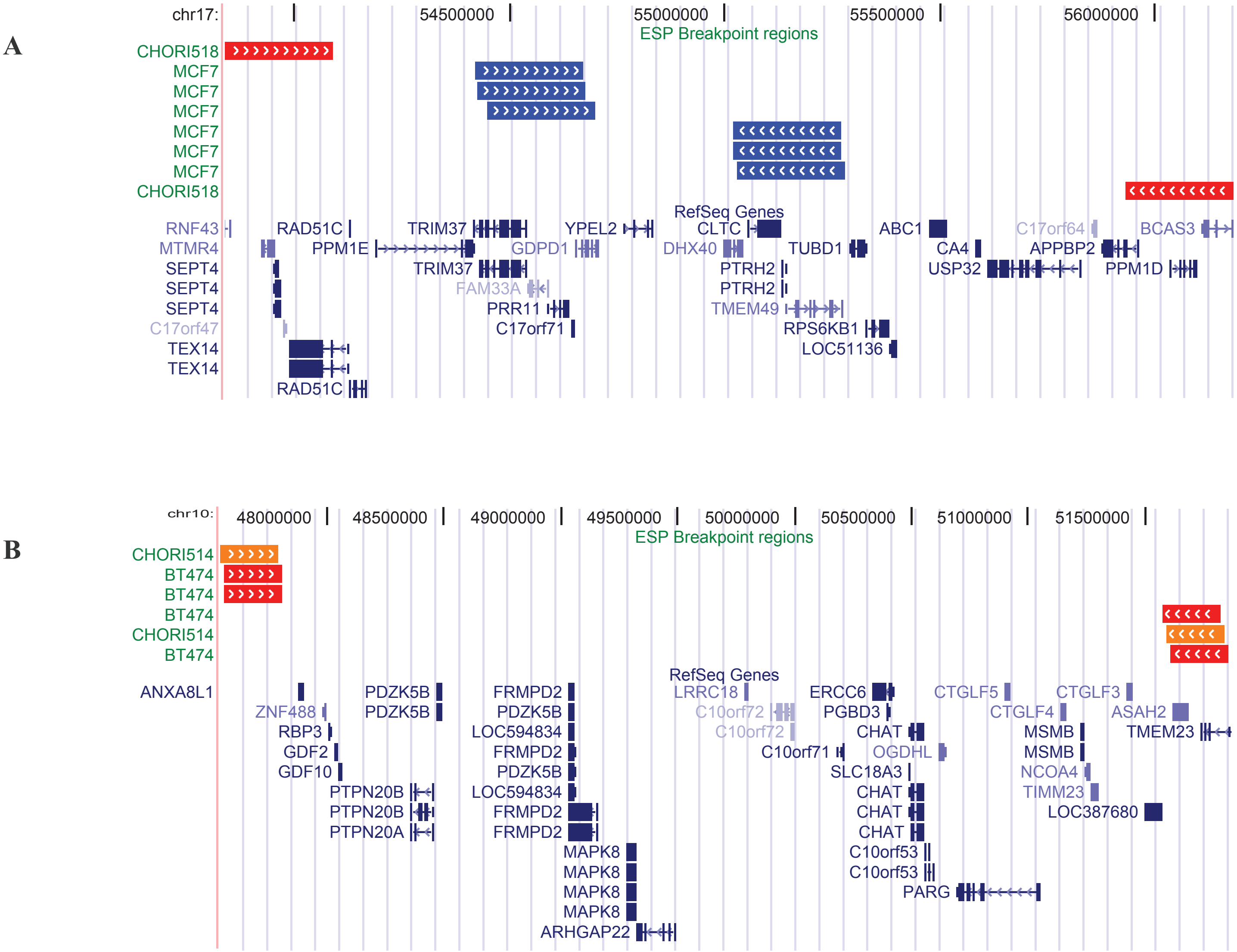

Figure S3: UCSC Genome Browser views of (A) An 820-kb deletion in 17q23.3 in MCF7 and BT474 that contains the TRIM37, GDPD1, YPEL2, DHX40, and CLTC genes. (B) A 4 Mb deletion of gene-rich region in 10q11.22-10q11.23 in BT474 and a primary breast tumor (CHORI514).
